# Gender-related aspects in occupational allergies – Secondary publication and update

**DOI:** 10.1186/s40413-017-0175-y

**Published:** 2017-12-27

**Authors:** Monika Raulf, Thomas Brüning, Erika Jensen-Jarolim, Vera van Kampen

**Affiliations:** 1 Research Institute for Prevention and Occupational Medicine of the German Social Accident Insurance, Institute of the Ruhr University Bochum (IPA), Bürkle-de-la-Camp-Platz 1, 44789 Bochum, Germany; 2 Messerli Research Institute, University of Veterinary Medicine Vienna, Vienna, Austria

**Keywords:** Allergy, Asthma, Dermatitis, Hypersensitivity pneumonitis, Gender, Occupational allergens, Occupational diseases, Skin diseases

## Abstract

For years occupational allergies have been among the most frequently recorded occupational diseases, and both the skin as well as the respiratory tract may be affected. An estimated 9 to 15% of adult asthma is (partially) caused by work-related exposure. Gender-specific differences in exposure cause different risks in the workplace which affect the health of employees. This also applies to exposure and working conditions when handling sensitizing working substances. The presented gender-specific analysis of the German documentation of confirmed occupational respiratory allergic diseases and occupational skin diseases reveals clear differences between men and women in the number of diseases and also in exposure conditions and working areas.

## Background

### Rationales for gender-specific consideration

It is undisputable that biological differences between men and women exist. In addition, there are role-specific differences, which are based on the fact that men and women are treated differently by society, despite legal equality. While biological gender (in the English usage “sex”) is fixed, the social roles “male” and “female”, which are termed “gender”, are culturally shaped and are also governed by respective social conditions [1, 2]. Gender-specific differences mean that people cannot be viewed independently from their living circumstances. This is also reflected in the working world. The occupational activities of men and women, the jobs they do, their respective working conditions, and how they are thus treated by society, are different. These differences can also affect the dangers and risks faced by men and women in occupational activities, and the way in which these are judged and prevented [2].

The European Agency for Safety and Health at Work (EU-OSHA) identifies the following differences, which are influenced by gender-specific aspects:

1. Women work in specific sectors and do specific types of work.
2. Women are underrepresented at supervisory and management levels.
3. Women balance dual responsibilities at work and home (in the family).
4. Women differ physically from men, although the differences in physical strength are often greater within the group of women than those between men and women.
5. Women perform work that is often wrongly assumed to be safe and easy.

To equalize these shortcomings, gender equality strategies (so called gender mainstreaming) are integrated into the EU Health and Safety Policy of the EU-OSHA. In this way, the EU-OSHA aims to highlight these differences between women and men in the workplace, and helps to improve safety and health in areas where women are most affected [2].

Gender issues are becoming more prominent because the proportion of women in the European labor market has increased significantly in the past 25 years. In Germany in 2001, 62% of women were employed, while in 2014 the number had risen to 73% [3]. However, it should not be overlooked that women’s participation in the labor market is still lower than men (increase from 76% in 2001 to 82% in 2014) and women often have shorter working hours due to part-time employment. In Germany in 2015, for example, 89% of men were fully employed compared to only 52% of women (Mikrozensus 2015) [4]. There are considerable differences in participation rates among EU member states [2]. The rate of employment and the average working time for women also depends to a large extent on whether children are present. Although mothers worked with 73% more than the EU average in 2013 in Germany, they were mostly employed part-time (66%). After a parental leave period, which is still mainly taken by women, job re-entry for women is partially associated with lower-skilled activities and lower social status. Women in particular over the age of 50 carry the additional burden of dedicated caring for sick, disabled or elderly relatives.

Although almost all professions in Germany can now be undertaken by women, there are still so-called “women’s professions” (horizontal segregation). Women are more likely to work in the public sector, in the social services, care and education sectors, as well as in sales or as office workers [2]. In 2010, 77% of the workforce in the health-care sector was female. Men more often work as machine operators in technical professions, crafts, construction, transport and mineral production. Men are prevalent in activities dealing with machines and products which are considered to be “heavy” or “difficult”. In the occupational group of unskilled workers, women often practice a job as a cleaner, while men are generally employed as a “worker”. In addition, there is vertical segregation in the labor market: men are more often engaged in activities and jobs which are located further up in the occupational hierarchy. More men than women are in managerial and senior positions (in Germany, more than two-thirds of management positions in 2010 were occupied by men). However, the proportion of women in management positions strongly depends on the proportion of women in the industry as a whole. On average, men earn more than women, even if the income is adjusted for the hours actually worked [2].

### Different exposure to hazards for men and women

The horizontal and vertical segregation in the labor market cause gender-specific differences in exposure to hazards and their health consequences.

It has been noted [2] that men more frequently have accidents and injuries at work compared to women, while women complain more frequently about upper limb disorders and stress. Major threats to respiratory health tract for women come from cleaning and sterilizing agents and protective gloves (if the latter are made of latex material with powder), as they were or still are used in health care facilities, as well as from dust in the textile and clothing industry. Women are at greater risk of suffering skin disease, for example, by working in humid conditions in the cleaning and hospitality branch and/or by skin contact with cleaning agents, or by the chemicals used for professional hairstyling. Especially women in the care and education sector suffer from infectious diseases more often. In contrast, men, due to exposure to noisy machines and equipment, are more likely to suffer from hearing impairment as a result of noise than women. Men often lift heavy loads and are injured by lifting and carrying. However, this also applies to women in the cleaning and care sector. Both women and men report high levels of work-related stress.

### Occupational allergic diseases

Occupational allergies have been one of the most frequently reported occupational diseases (BK, Berufskrankheiten) for years [5]. In Germany, according to the ordinance of occupational disease the following occupational allergic diseases are recorded (Table 1): “Obstructive airway diseases (including rhinopathy) caused by allergic substances”, Occupational disease no. 4301 (BK 4301) as well as “Hypersensitivity pneumonitis” (HP), Occupational disease no. 4201 (BK 4201). Obstructive airways and HP disorders caused by “isocyanates” are documented separately under the BK 1315. While obstructive airway diseases caused by irritant and toxic substances are summarized under BK 4302 and are therefore distinguished from the BK 4301, allergic as well as non-allergic “severe or repeatedly recurrent skin diseases” are subsumed together under the BK 5101, also including the isocyanate-induced skin diseases. Due to this lack of differentiation in the records, a specific classification of cases of allergic and non-allergic skin diseases is not possible. The most frequent form of occupational skin disease is the so-called acute-irritant contact dermatitis, resulting from direct exposure to acids, alkalis or other aggressive chemicals. The second most common work-related skin disease is allergic contact dermatitis. In many patients, however, a synergistic combination of irritative and allergic mechanisms is present [6], therefore, a clear distinction based on the pathomechanism is difficult.

**Table 1:** Occupational allergic diseases (according to the list of German and Austrian occupational diseases)

Occupational disease no. (BK no.)	Skin and airway diseases*^1^	Austrian § 177; ASVG-list*^2^
1315	Diseases induced by isocyanates, forced to refrain professional activities which are responsible or may be responsible for the induction, aggravation or resurgence of the disease	__
4201	Hypersensitivity pneumonitis (inflammatory alterations of the pulmonary alveoli)	43
4301	Obstructive airway disease induced by allergenic substances (including rhinopathy), forced to refrain from professional activities which are responsible or may be responsible for the induction, aggravation or resurgence of the disease	30
5101	Severe or repeatedly recurrent skin disease, forced to refrain from professional activities which are responsible or may be responsible for the induction, aggravation or resurgence of the disease	19
__	Allergy induced anaphylactic reactions after latex sensitization*^3^	53

*^1^ Translation of the German BK-list

*^2^ Allocation was done by analogy, but the wording is still not identical

*^3^ The listed wording corresponds to the list of occupational diseases according to § 177 of the Austrian General Social Insurance Law (ASVG)

In principle, only those diseases caused by special exposure on the job can be recognized as occupational diseases, according to medical science knowledge. Certain groups of subjects are exposed to harmful substances by their occupational activities to a considerably higher degree than the rest of the population. For some diseases, confirming that there is an occupational cause depends on additional insurance prerequisites. For the BK nos. 5101, 4301 and 1315, conditions are only recognized as occupational diseases if the affected person refrains from all activities which led or could lead to the development, aggravation or recurrence of the illness. If such conditions are not fulfilled, formal occupational disease recognition is not possible. Nevertheless, extensive benefits for prevention, curative treatment and vocational help are often granted in these cases.

The current data that follow on the four above mentioned occupational diseases from the commercial, public, and agricultural sectors in Germany, which cover allergic symptoms, are presented in terms of gender-specific differences.

## Methods

Occupational diseases as well as work, school and road accidents, which are reported to statutory trade associations and accident insurance companies, are documented and published by the German Statutory Accident Insurance (DGUV) within the documentation of occupational disease (BK-DOK) http://publikationen.dguv.de/dguv/pdf/10002/12640neu.pdf. Farmers, however, are not insured through the DGUV, they are insured through the Social Insurance for Agriculture, Forestry and Horticulture (SVLFG).

The data for the subsequent evaluation were made available to us by the DGUV and include the number of all confirmed cases of occupational diseases (BK) and especially the occupational diseases nos. 1315, 4201, 4301 and 5101 between 2010 and 2014, differentiated by sex. For the same period, the number of all confirmed cases of occupational disease from the agricultural sector and the confirmed cases of BK 4201, BK 4301 and BK 5101 were also made available for us from the SVLFG (cases of a BK 1315 were not available here). Confirmed occupational diseases include cases which meet the medical characteristics of an occupational disease and the determination of an occupational cause, but which are independent of the insurance requirements for the recognition of an occupational disease, such as stopping the harmful job activities. However, comprehensive benefits of prevention and rehabilitation are provided for the insured worker. In this evaluation especially the "specific object for the occupational disease", i.e., the disease-inducing hazard triggers, were investigated. Triggers were grouped into categories and summarized to generic terms. The data from the areas of the DGUV and the SVLFG were evaluated together in a descriptive manner.

Statistical analyses of differences between male and female distribution among the different occupational diseases by two-sided Fisher’s exact test were performed with GraphPad Prism software (GraphPad Software, San Diego, Calif, USA) and a significance level of 0.05 was chosen for all tests.

## Results

**Fig. 1:**
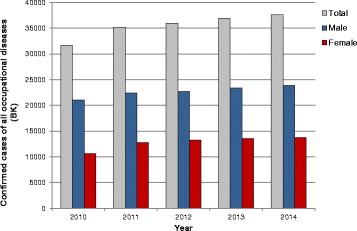
Numbers of all confirmed cases of occupational diseases as documented from the DGUV and SVLFG in Germany between 2010 and 2014

**Fig. 2:**
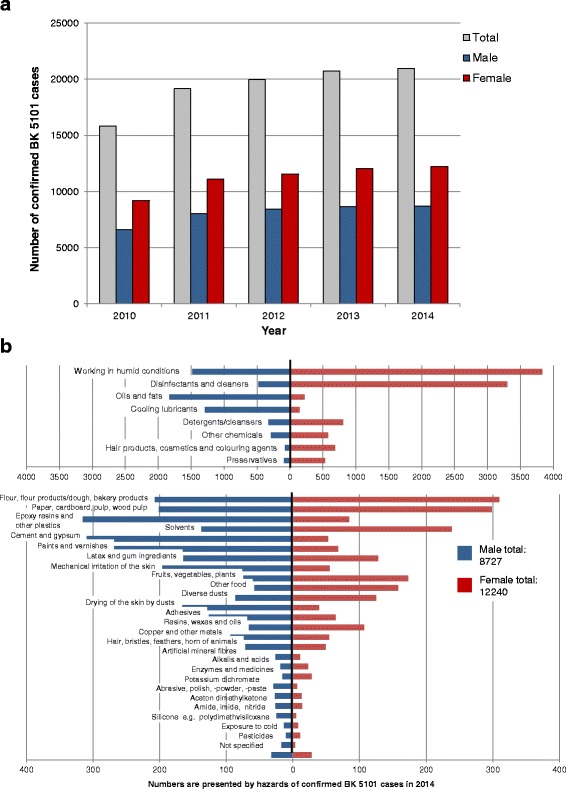
Numbers (**a**) and triggers (**b**) of confirmed cases of occupational diseases (BK) 5101 (skin diseases) as documented from the DGUV and SVLFG

Between 2010 and 2014 the total numbers of confirmed occupational diseases in Germany increased from 31,691 in 2010 to 37,621 in 2014 (Fig. 1). The majority of the confirmed cases (about 98%) originate from industrial sector reported by the DGUV and only 2% from the agricultural sector (SVLFG). Classification of the confirmed of occupational disease cases according to gender shows that 63% men and 37% women were affected (average over the period of five years) (Table 2). Among the occupational diseases presented here, BK 5101 (severe or repeatedly recurrent skin disease) is represented the most (on average over the five year period 55% of all confirmed occupational diseases), with a total of 20,721 confirmed cases in 2014, which increased between 2010 and 2014 (Fig. 2a). Only a very small proportion of the confirmed cases of BK 5101 (in total between 8 to 17 cases) originate from the agricultural sector (SVLFG). The classification according to gender (Table 2) shows that on average over the five years investigated, 42% men and 58% women were affected by this disease. On average 87% of all confirmed occupational diseases among women (e.g. 13,742 cases in 2014) are related to the BK 5101 (12,019 cases in 2014). In contrast only 36% of all confirmed occupational diseases among men are related to this occupational disease (8702 cases from 23,879 in 2014) (Table 2). Working in humid conditions is listed as the most frequent cause for both genders (women *n* = 3828, men *n* = 1438) in 2014, as in the previous years, followed by disinfectants and cleaners in women (Fig. 2b). Additionally, detergents/cleansers as well as hair products, cosmetics and colouring agents are more frequently listed for women as triggers of a BK 5101 than for men. Cooling lubricants as well as oils and fats represent a large proportion of skin disease triggers in men. Of the total number of 528 skin diseases affected by flour, flour products, dough and bakery products in 2014, 59% of the affected were women. Comparably high is also the percentage of women (60%) for the trigger “paper, cardboard, pulp, wood pulp”. In the categories of “fruit, vegetables, plants” and “other food” the percentage of affected women is 70 and 73%, respectively, significantly higher than the proportion of men.

**Table 2:** Occupational allergic diseases – classification of the confirmed cases according to gender – average over 5 years (2010–2014)

	Percentage [%] among the specific disease		Percentage [%] of all confirmed occupational diseases among	
	male	female	male [100% = 22,725]	female [100% = 13,232]
All confirmed occupational diseases [100% = 35,957]	63	37***	–	–
BK 5101	42	58***	36	87***
BK 4301	63	37***	1.87	1.85
BK 1315	81	19***	0.15	0.06*
BK 4201	69	31***	0.18	0.12

Occupational disease (BK) 5101: skin diseases, BK 4301: allergic obstructive respiratory diseases, BK 1315;

isocyanates, BK 4201: hypersensitivity pneumonitis; Significant differences between male and female: * = *p* < 0.05; *** = *p* < 0.0001

**Fig. 3:**
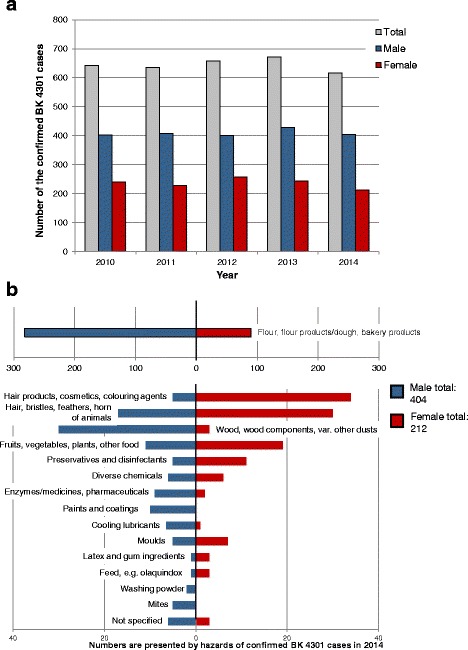
Numbers (**a**) and triggers (**b**) of confirmed cases of occupational diseases (BK) 4301 (allergic obstructive respiratory diseases) as documented from the DGUV and SVLFG

Between 616 and 672 cases per year of BK 4301 ("Obstructive airway diseases, including rhinopathy, caused by allergic substances") were confirmed from 2010 to 2014 (Fig. 3a), representing on average 1.85% of all confirmed cases of occupational diseases. The average proportion of men was 63% and that of women 37% (Table 2). In relation to the total number of confirmed occupational diseases among men only 1.87% is related to BK 4301. Nearly the same frequency (1.85%) of confirmed occupational diseases among women was related to this disease (Table 2). Flour, flour products, dough/bakery products (typical triggers of “baker’s asthma”) are the most common category of triggers at more than 60% (Fig. 3b). For significantly more men than women, these triggers are the cause of an obstructive airway disease according to BK 4301 (76% versus 24%). Further typical triggers of a BK 4301 can be found in the hairdressing and cosmetics sector. As expected, in this sector the proportion of women is significantly higher than in men (87%). Also significantly more women are affected by "hair, bristles, feathers, horn of animals" (81%), while the application of “wood, wood components and various other dusts” was more often confirmed as the cause of respiratory allergy in men (91%). Similar to BK 5101, the triggers of cooling lubricants, paints and coatings as well as enzymes/medicines and pharmaceuticals significantly affected more men (Fig. 3b). More women (69%) suffer from allergic asthma or an allergic rhinopathy induced by exposure to preservatives and disinfectants. In addition to the work sectors “foodstuffs industry and catering trade”, the reports of an obstructive airway disease triggered by an allergen come from sectors like health and welfare services, metalworking and wood processing, trade and administration, waste and waste recycling, and agriculture with grain cultivation and animal farming.

**Fig. 4:**
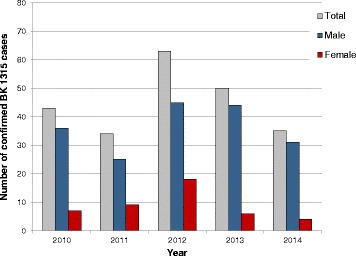
Numbers of confirmed cases of occupational diseases (BK) 1315 (isocyanate diseases) as documented from the DGUV and SVLFG

Between 34 and 63 cases of BK 1315 were confirmed in the years from 2010 to 2014 (Fig. 4), representing on average 0.12% of all confirmed cases of occupational disease. All cases come from the industrial sector reported by the DGUV. The BK 1315 covers all respiratory diseases caused by isocyanates. Isocyanates have broad applications in the production of soft, hard, integral, insulating and foaming materials and other plastics, paints and other surface coatings, potting compounds, elastomers, adhesives, hardeners, pharmaceuticals, pesticides and other chemical products. The main areas of application are the automotive, aircraft, metal, furniture and woodworking industries, construction, mining, foundries, textile and clothing manufacturing and sports track construction [7, 8]. As expected, BK 1315 was confirmed only in a very small proportion of women (average 19% of all confirmed BK 1315) and only 0.06% of all confirmed occupational diseases among women (Table 2). In relation to all confirmed occupational diseases among men, 0.15% was related to isocyanates.

**Fig. 5:**
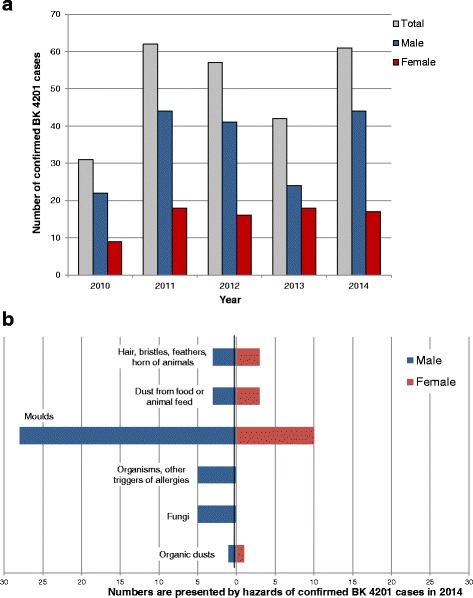
Numbers (**a**) and triggers (**b**) of confirmed cases of occupational diseases (BK) 4201 (hypersensitivity pneumonitis) as documented from the DGUV and SVLFG

Another - rather rare - allergic disease is BK 4201 (HP). Between 2010 and 2014 an increase of the confirmed cases of BK 4201 can be observed, whereby on average only about 50 BK-cases per year were confirmed (Fig. 5a). Calculated over the five years, the average percentage of women with confirmed BK 4201 was 31% (Table 2). Only 0.18% of all confirmed occupational diseases among men (Table 2) were related to this disease. The percentage among women was a bit smaller (0.12%) but no significant difference existed (Table 2). Almost 70% of the confirmed BK 4201 cases originated from the agricultural sector, because moulds/fungi and components of organic dusts (including micro-organisms) as well as hair, bristles, feathers, horn of animals were most frequently mentioned as occupational triggers for this occupational HP (Fig. 5b).

## Discussion

The specific evaluation of the BK-DOK of the DGUV as well as of the statistics of the agricultural sector (SVLFG) for work-related allergic diseases differentiated by sex clearly shows that in Germany occupational diseases of the respiratory tract are more prevalent in men (BK 4301, BK 1315 and BK 4201), while occupational skin diseases (BK 5101) are more prevalent in women. The classification of confirmed cases based on the underlying trigger suggests that specific exposures on-the-job or the handling with some agents during the worktask are more typical for women than for men or vice versa. Although the data of our analyses is based on differentiation by sex, in turn, they reflect the gender-specific differences in choice of profession.

Occupational skin diseases are the second most common occupational disease worldwide. Fifty-five percent (55%) of all confirmed occupational diseases in Germany are related to this occupational skin disease (BK 5101) with a significantly higher proportion of all confirmed occupational diseases among women (87%). Occupational contact dermatitis (OCD) is the most frequent occupational skin disease, and comprises irritant contact dermatitis, allergic contact dermatitis, contact urticaria and protein contact dermatitis [9]. The incidence of occupational skin diseases in western industrial countries is estimated at 0.5–1.9 cases/1000 occupants/year, but it is assumed that the prevalence of OCD is underestimated by a factor of 30–50 [10]. OCD is associated with a high socioeconomic burden based on sick leave, lost productivity, dermatological treatment, vocational retraining and workers compensation causing high costs and severe economic implications for companies and social security systems. There are many endogenous and exogenous factors which affect the development of OCD, including age, sex, ethnicity, atopic skin diathesis, certain occupations and environmental factors [9]. One of the most important contributing causes is skin barrier dysfunction. High risk occupations for OCD include health care workers, hairdressers and construction workers. There are often multiple contributing causes to OCD, as workers are exposed to both irritants and allergens.

When considering BK 5101, it must be taken into account that this occupational disease number covers both allergic and irritant-induced skin diseases. Therefore an isolated consideration of the allergic reactions is not possible. On the other hand, as mentioned before an unequivocal differentiation of skin diseases of allergic or irritative genesis is not always possible, because it is easier to develop allergic contact dermatitis on irritated or damaged skin than on intact skin. Both the literature [11] and our data show that wet work/humid conditions is the main irritant for both genders, which can lead to preliminary damage of the skin. As it is, the term “working in humid conditions” includes both activities in which employees regularly work with their hands in wet environments and the prolonged wearing of protective gloves [12]. Here, not only cleaning staff but also the “traditional female occupations”, like hairdressers and nurses, are affected. This explains the extremely high numbers of occupational skin diseases among women [13]. Although especially in the health care sector for hygiene reasons the use of disinfectants and protective gloves is steadily increasing, the numbers of the confirmed BK 5101 cases have remained unchanged in the last two years of reporting, which may be attributable to focused skin protection and prevention campaigns. For men, the situation is more heterogeneous and the occupational diseases of the skin extend to a wider range of triggers, such as cooling lubricants, oils and fats, which frequently affect the professional group of metal workers.

To what extent gender-specific behaviors, such as different skin care and cleaning, affect the numbers of occupational skin diseases cannot be obtained from the analyzed data, but the implications seem obvious. For example, a study of more than 1000 metalworkers shows that significantly more female than male workers use skin protection cream at work — but also at home [14]. Furthermore, it is likely that there are gender differences in how quickly someone consults a doctor after the occurrence of skin alterations. According to a study in Germany, women show a higher sensitivity to their body and health, as well as a greater willingness to accept medical help. Men often consult a doctor only after a disease has manifested, whereas women consult a doctor earlier, when the first symptoms appear [15]. This probably applies for dealing with skin as well as for allergic respiratory diseases.

In the last five years almost twice as many men as women were affected by occupational allergic obstructive airway diseases. Flour and flour products, as well as wood, wood components and various other dusts are the dominate triggers for men. Women are also mainly affected by flour and flour products, followed by hairdressing products, cosmetics and dyes, animal hair, bristles, and so forth. A similar distribution was also found by Latza et al. 2007 [16] when evaluating occupational allergic and irritative obstructive airway diseases in the commercial sector for 2004. The most affected occupations among men were bakers, carpenters, painters, whereas among women they were bakers, hairdressers, and employees handling laboratory or farm animals. Overall, it is striking that especially in the baker’s trade both women and men are at relatively high risk of developing occupational skin and respiratory diseases. Despite these similarities, gender differences are also found here. Male bakers are almost exclusively engaged in activities with high flour dust exposure such as bread baking, while female bakers mainly perform activities in the confectionery and pastry sector which are associated with lower exposure [16].

Microorganisms and their components belong to the main triggers of the relatively rare occupational HP disease [17], therefore considering the number of diseases reported in the agricultural sector was essential. In fact, 70% of the HP cases were documented by the SVLFG, while for the other three occupational diseases the majority of cases was recorded by the DGUV. Women are affected in only 30% of HP cases, which is probably due to the unequal involvement of women and men in farming. In 2003, 44% of men working in agriculture were active farm owners. In contrast, only 7% of women were owners, but 57% were family members supporting the work in the family enterprise (mainly the wife of the farmer). The proportion of these family members engaged in part-time work was around 75%. In spite of the relatively high level of family-member support, women play a subordinated role in the employment rate in agriculture. Obviously, they present additional resources for support in the family enterprise when necessary, but they are not extensively involved in the regular work [18].

### International studies

Various studies, as well as the summary representation of EU-OSHA [2], investigated the extent to which gender-specific differences in working conditions result in different health risks depending on country, and their consequences for health. Thus, for asthma and other respiratory diseases, men are noticeably more affected than women throughout Europe as a whole. In the EU labour force survey, 8.4% of men and 6.4% of women reported respiratory or pulmonary complaints as the most serious work-related health problems. In Luxembourg, Portugal and the United Kingdom, on the other hand, the proportion of women was higher [2]. In many other EU countries, health care, textile industry, food production, and the hairdressing sector are considered predominantly women’s activities, in which the most frequent causes of work-related asthma can be found. Likewise in other EU countries, skin diseases — triggered by frequent work in humid conditions — are typical diseases of the female-dominated health care professions or the cleaning sector [2]. Domestic workers and persons active in the household are continuously disregarded in the directives for occupational health and safety, and also in the occupational disease statistics. These workers particularly include women, who also support the mostly male partner in small family-run enterprises (e.g., in agriculture), and are thereby exposed to hazards.

At this point, it should be mentioned that comparing occupational disease data from different countries poses problems. Economic structures, legal frameworks, preventive measures and the documentation of occupational diseases differ between countries. There are often no specific incidences and it is usually assumed that the risks are underestimated [19]. It is also important to take into account that in many countries “asthma” is used as a general term, whereas in Germany according to the ordinance of occupational diseases “obstructive airway diseases” are recorded and, depending on the triggering substances assigned to two different BK numbers (allergic versus irritative). For example, BK 4301, which has been evaluated here, includes allergic obstructive airway diseases (including rhinopathy) and BK 4302 irritant or toxic obstructive airway diseases.

In a meta-analysis of the effect of occupational exposure on gender-specific differences in respiratory health, 12 occupational health studies including 3011 workers in “organic dust” industries (food processing workers, textile workers and farmers) concluded that female workers had significantly less chronic cough, chronic phlegm as well as chronic bronchitis than their male coworkers after the adjustments for smoking, age and duration of employment [20]. Upper respiratory tract symptoms by contrast were more frequent in women than in men in these groups. Significant gender related lung function differences occurred in the textile industry but not in the food processing industry or among farmers. The authors pointed out that in this study it cannot be determined whether these findings represent true physiological gender differences, gender specific workplace exposures, or other undefined gender variables.

An additional meta-analysis by Dimich-Ward et al., [21] including data on 1367 women and 4240 men showed that women have a higher risk of shortness of breath compared to men, and that the type of exposure (e.g., inorganic versus organic) strongly influences the extent of gender-specific differences. For example, among workers exposed to inorganic dusts, the risk of recurrent asthma was extremely high for women relative to men; in contrast to this no gender differences could be found for unexposed workers. The authors speculated that the high risk of asthma in women may be a result of asthmatic men avoiding employment in more highly exposed jobs. Gender-specific differences in occupational asthma were also confirmed in a study by White and co-workers [22], which analyzed data from the monitoring program in the US states of California, Massachusetts, Michigan and New Jersey during the period 1993–2008. This showed that women are more likely to be affected by occupational asthma in health care, education and services, as well as in sales and administration than men. However, the evaluation also verified that in total men developed asthma for the first time during their work, whereas women had asthma, but the symptoms exacerbated during job exposure.

In a recent study by Schyllert et al. [23] based on re-examination of three population-based cohorts in the “Obstructive Lung disease in Northern Sweden” (OLIN) the authors did not find a relationship between dust exposure and asthma among women, while inorganic dust increased the risk of asthma among men. Furthermore, exposure to chemicals increased the risk of rhinitis only among men but not among women. The authors were not able to establish whether these sex differences are a result of biological differences or due to exposure to different substances or chemicals.

Additionally, evaluation of data from the longitudinal Northern European population study [24] demonstrated that new-onset and respiratory symptoms increased in women as they became postmenopausal. One must keep in mind that menopause is associated with profound hormonal and metabolic changes. These may also influence the respiratory health via female and male sex hormones acting differently on resident lung cells and immune function. Therefore, these findings should be taken into account in occupational safety and health strategies especially for older female but also male workers.

## Conclusion

Overall, allergic skin and respiratory diseases that are triggered or exacerbated by working activities play an important role in Germany as well as in other EU countries, both for women and for men (in Germany on average 87% and 36%, respectively). Due to the gender-specific segregation, women and men are exposed to different exposures in the workplace. However, not just sex per se, but differences in working conditions, behaviours, and also in health consciousness are essentially responsible for health problems in the workplace. In future studies which intend to contribute to a more accurate risk assessment and improved health care in the working environment, a simple gender comparison is not enough. A multivariate model should be used to study which protective and risk factors are important in the workplace, and whether they differ with gender. Based on different health awareness in men and women, workplace health promotion strategies need different approaches for male and female audiences. In general, health in the workplace should not be a question of gender.
